# Improvement of Oil and Water Barrier Properties of Food Packaging Paper by Coating with Microcrystalline Wax Emulsion

**DOI:** 10.3390/polym14091786

**Published:** 2022-04-27

**Authors:** Dongyang Liu, Yuqing Duan, Shumei Wang, Murong Gong, Hongqi Dai

**Affiliations:** Jiangsu Co-Innovation Center of Efficient Processing and Utilization of Forest Resources, Nanjing Forestry University, Nanjing 210037, China; dyliu@njfu.edu.cn (D.L.); duanyuqing1228@163.com (Y.D.); wsm@njfu.edu.cn (S.W.); gongpy@njfu.edu.cn (M.G.)

**Keywords:** microcrystalline wax emulsion, oil and water resistance, fluorine-free oil repellent

## Abstract

Studies have shown that fluorinated oil repellents are potentially harmful to humans and the environment, and therefore, the development of non-toxic, green, and environmentally friendly oil repellents has become inevitable. Microcrystalline wax is a branched saturated alkane with a molecular weight of 580–700 Da, which has a lower surface tension than edible oil. Herein, microcrystalline wax emulsion (fluorine-free oil repellent) was prepared by mechanical stirring–homogenization, the effects of emulsifier ratio and dosage on the emulsion performance were systematically investigated, and the resultant stable microcrystalline wax emulsions were applied to the paper surface to explore the oil and water resistance and water vapor barrier performance. The results showed that stabilized microcrystalline wax emulsion was obtained at the emulsifier Span-80/Tween-80 ratio of 5:5, and the emulsifier dosage was 20% (relative to the microcrystalline wax). When 6 g/m^2^ of microcrystalline wax was applied to the surface of starch pretreated paper, the kit rating value of the paper was high, at up to 10/12, the Cobb_60_ value decreased to 12.5 g/m^2^, the overall migration of paper was less than 10 mg/dm^2^, and the water vapor permeability was reduced by 81.9%, which met the requirements of oil and water resistance performance of food packaging paper.

## 1. Introduction

Owing to its ability to create an extreme wettability surface [1,2], fluorinated polymers are widely used in food packaging, such as sandwich wrappers, pizza boxes, cardboard cartons, etc., [3,4,5,6]. Long-chain fluorinated polymers (type C8) are easily decomposed to form new compounds at high temperature, such as perfluorooctanoic acid (PFOA) and perfluorooctane sulfonate (PFOS), which are difficult to degrade and will cause various chronic diseases when accumulated in the human body [7,8,9]. As early as 2006, the European Union issued the 2006/122/ECOF decree, which banned the use of PFOS and restricted the use of PFOA and its salts [4]. Therefore, it has become an inevitable trend to develop non-toxic, green, and environment-friendly fluorine-free oil repellents. With the advantages of being widely available, renewable, and environment-friendly, the development of biomass oil repellents has been a high-interest topic for several years [10,11,12,13,14]. However, the widespread adoption of these materials for practical applications is still a challenge due to the relatively high costs of biomass oil repellents, in addition to the poor resistance of these materials to water.

Wax is widely used as a material for food packaging, fruit preservation material, etc., [15,16]. Because of its excellent water resistance, wax emulsions have generally been used as a water repellant in the paper-making industry [17]. Han et al. [18] applied whey protein isolate (WPI)/cellulose-based films with beeswax (BW) onto paperboard by heating compression, which had a strong barrier effect with a decrease in water vapor transmission rate (WVTR) to 92−95%. Zhang et al. [19] applied polyhexamethylene guanidine hydrochloride (PHGH) and polyhexamethylene biguanide (PHMB) modified beeswax and carnauba wax emulsion to copy paper, which, compared with the blank sample, the WVTR of the paper with a coating load of 12 g/m^2^ was reduced significantly from 2788.8 g/m^2^/d to 222.0 g/m^2^/d. Khwaldia [20] successfully fabricated a type of packaging paper with degradable moisture-resistant coating, where the components of sodium caseinate (NaCAS) and paraffin wax in the coating played the role of reducing the WVTR of the paper. Naderizadeh et al. [21] prepared a superhydrophobic coating by using a wax emulsion made from a copolymer of beeswax and perfluorinated acrylic acid. Zhang et al. [22] successfully created a superhydrophobic surface with a low sliding angle by coating the paper surface with a wax mixture (beeswax/carnauba wax) emulsion followed by an annealing treatment. To date, fewer studies have been reported on wax being developed as oil repellent for paper. Microcrystalline wax with a melting point of 60 °C to 90 °C is ductile and non-fragile at low temperatures, and can be used as chewing gum additives, packaging impregnation, fruit coatings, etc., [23,24,25]. The substance was evaluated by the Scientific Committee on Food (SCF) and the Joint FAO/WHO Expert Committee on Food Additives (JECFA) in 1995. They concluded that there was no concern for genotoxicity from microcrystalline wax [26]. Therefore, the use of microcrystalline wax as a paper coating does not pose a food safety concern.

**Figure 1:**
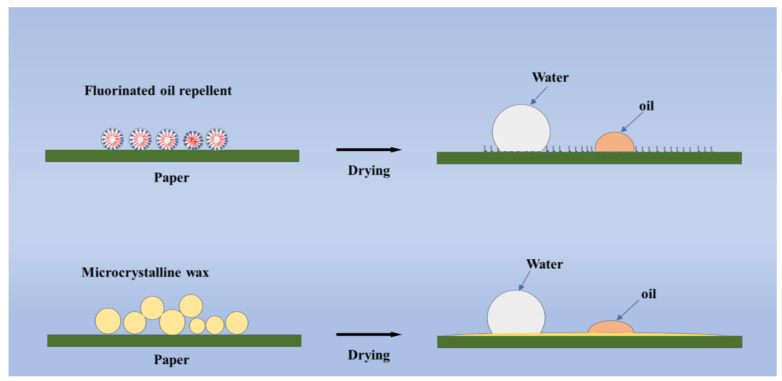
Oil and water resistance mechanism of fluorinated oil repellents and microcrystalline wax.

In this study, microcrystalline wax was emulsified by mechanical stirring and high-pressure homogenization, and a stable microcrystalline wax emulsion was produced. The effects of emulsifier ratio and dosage on emulsion performance were investigated. Furthermore, the oil and water resistance, and the water vapor barrier properties of the paper coated with the above emulsion were systematically studied. Unlike fluorinated oil repellent that changes the surface energy of paper, microcrystalline wax can form a uniform barrier layer on the paper surface, thus achieving the goal of oil and water resistance; this mechanism is shown in Figure 1. Overall, this study puts forward a feasible and green approach to replacing fluorine oil repellent with microcrystalline wax emulsion as an oil repellent for paper.

## 2. Materials and Methods

### 2.1. Materials

Microcrystalline wax (80#, food grade) was purchased from Weijia Industrial Co., Ltd. (Jingmen, China). Emulsifier Span-80 and Tween-80, both pharmaceutical grade, were purchased from Aladdin Biochemical Technology Co., Ltd. (Shanghai, China). Castor oil (chemically pure) was purchased from Lingfeng Chemical Reagent Co., Ltd. (Shanghai, China). Original paper (grammage 70 g/m^2^, kit rating 0/12) was purchased from Jinguang Chuangli Office Paper Co., Ltd. (Shanghai, China). Cationic starch (substitution degree of 0.025~0.03) was purchased from Hefei BASF Biotechnology Co., Ltd. (Hefei, China). According to CODEX STAN 192-1995 General Standard for Food Additives, the above-mentioned emulsifiers can be used in food production.

### 2.2. Experimental Methods

#### 2.2.1. Preparation of Microcrystalline Wax Emulsion

An amount of 20 g of microcrystalline wax was placed in a three-neck flask and heated to melting point, and then 2–7 g of the compound emulsifier was mixed with the molten microcrystalline wax while keeping the three-neck flask at 95 °C. Subsequently, an appropriate amount of deionized water was gradually added to the three-neck flask under high-speed stirring to keep the solid content of the mixture at about 30%. After thorough stirring, the obtained mixture was subjected to high-pressure homogenization twice at a pressure of 400 bar, and the microcrystalline wax emulsion was obtained after rapid cooling. The compound emulsifier consisted of Span-80 and Tween-80 in the ratios of 3:7, 4:6, 5:5, 6:4 and 7:3.

#### 2.2.2. Original Paper Pretreatment

The cationic starch solution with a concentration of 5 wt% was prepared and then applied to the surface of the original paper with a coating load of 1.5 g/m^2^. The coated paper was dried in the drying oven (DGG-9070A, SENXIN, Shanghai, China) at 105 °C for further processing.

#### 2.2.3. Microcrystalline Wax Emulsion Surface Coating

The coatings were applied on the paper’s surface using the manual coating method. For each coating, a 3.0 mL of microcrystalline wax emulsion with different concentrations was applied to the paper surface and then dried in the drying oven at 80 °C. A total of 18 batches of paper samples were produced under different conditions. Twelve sheets of each sample were produced for further studies. Additionally, these paper samples were treated at 23 °C under 50% relative humidity before performing further studies.

#### 2.2.4. Emulsion Property Testing

Particle size: The particle size of microcrystalline wax emulsions was measured using a nano-laser particle size analyzer (Zetasizer Nano ZS, Malvern, UK) in the range of 0.3 nm–100 µm. Before measurement, the samples were prepared by diluting the emulsion nearly 300 times with deionized water. There were three tests for each sample.

Stability: An amount of 10 mL of the emulsion was injected into the centrifuge tube and centrifuged at 4000 r/min for 15 min, and then observed as to whether the emulsion stratified. If the emulsion was stratified, it meant that the emulsion was not stable; if not, it meant that the emulsion was stable.

Viscosity: The apparent viscosity of the microcrystalline wax emulsion was measured using a rotary viscometer (NDJ-79, Shanghai Precision Instrumentation Co., Ltd, Shanghai, Shanghai, China) in the range of 1–1 × 10^6^ mPa·s. Three measurements were taken of each sample and the mean values were reported.

#### 2.2.5. Oil and Water Resistance Testing of Paper

Oil/Grease Resistance (Kit Rating): Uncoated paper and coated paper were tested to evaluate their grease resistance following the TAPPI standard T559pm-96 protocol [27]. Five measurements were taken of each sample and the mean values were reported.

Water Resistance (Cobb_60_): Water absorption was adopted to determine the water resistance of the paper. In detail, water absorption was tested by using the Cobb tester (P95930 Z000, PTI, Birkenau, Germany) according to standard tests ISO 535, and was expressed as a Cobb_60_ value (g/m^2^), which refers to the mass in grams of water that was absorbed per square meter of paper material.

Advancing and Receding Contact Angles (*θ_a_*/*θ_r_*): To determine the resistance of the sample to the penetration of water and grease, an optical contact angle tester (T200-Auto3 Plus, Baiorin, Sweden) was employed for contact angle determination. An amount of 4 μL liquid droplets (water or castor oil) were placed on the surface of the paper samples, then the sample surface was slowly tilted, and the advancing contact angle (*θ_a_*) and receding contact angle (*θ_r_*) were recorded when the droplets were about to move. Each sample was measured three times.

#### 2.2.6. Scanning Electron Microscopy (SEM)

The micrographs of the original paper and coated paper were recorded with SEM (Regulus 8100, Hitachi, Japan). Samples were prepared for SEM characterization by attaching them to aluminum columns using carbon double-sided tape and then spraying them with gold.

#### 2.2.7. Water Vapor Permeability (WVP)

Based on the method described in ASTM E96-05, the WVP was tested on original paper and coated paper using a water vapor permeability tester (W3/060, labthink, Jinan, China) at a temperature of 38 °C and 70% relative humidity. Each sample was tested three times.

#### 2.2.8. The Overall Migration of Paper

Migration tests were conducted on the coated paper according to the migration test conditions in SN/T 3044-2011 “Food contact material for export–Polymeric coatings on paper and board–Guide to the selection of conditions and test methods for overall migration”. In this paper, 95% (*v*/*v*) ethanol solution was used as a grease food simulant. A 10 cm × 10 cm specimen was immersed in 200 mL of the food simulant and treated at 40 °C for 24 h to investigate the overall migration of the paper in the food simulant. Each sample was tested three times. The overall migration was calculated as Equation (1):

(1) $$m=\frac{m_{a}-m_{b}}{S}$$

where *m* (mg/dm^2^) is the overall migration, *m_a_* (mg) is the mass of extracted solvent residue after evaporation after the extraction test, *m_b_* (mg) is the mass of extracted solvent residue in the blank test, and *S* (dm^2^) is the contact area between the paper and the food simulant.

#### 2.2.9. Statistical Analysis

A statistical analysis of data was performed through a one-way analysis of variance using Statistical Analysis System (SPSS), version 26, and differences among mean values were processed by the Tukey test. Significance was defined at *p* < 0.05.

## 3. Results and Discussion

### 3.1. Factors Influencing the Preparation of Microcrystalline Wax Emulsions

Many factors affect the properties of emulsions, such as the type and ratio of emulsifiers, the emulsifiers’ dosage, emulsification temperature, emulsification mode, emulsion solid content, emulsion viscosity, etc. This study focuses on the effect of the emulsifier ratio and the emulsifier dosage on the emulsion performance.

(a) Emulsifier ratio

It is obvious that emulsifier plays an important role in the preparation and stability of emulsions [28]. To achieve higher droplet stability, combinations of hydrophilic and lipophilic emulsifiers are often used, which are thought to be compatible with each other and give more stiffness and strength to the emulsifier film. In this study, the effect of emulsifier ratio on the property of emulsions was investigated using a combination of lipophilic emulsifier Span-80 and hydrophilic emulsifier Tween-80. The experimental results are shown in Table 1. The stability of the emulsion is usually related to the average size of the droplets, so the droplet size was measured. With the gradual decrease in the HLB value of the compounded emulsifier, the average particle size of the emulsion showed a phenomenon of first decreasing and then increasing [29,30]. The droplet size results were consistent with the stability of the emulsions, which showed excellent stability when the ratio of Span-80 to Tween-80 was 5:5, with the smallest average particle size of 304.8 nm.

(b) Emulsifier dosage

The dosage of the emulsifiers is the key to the formation of stable monolayers and bilayers on the surface of the particles [31,32]. When at a low dosage of the emulsifier, the dispersed microcrystalline wax particles cannot be fully wrapped by the emulsifier, resulting in unstable emulsions. However, the excessive addition of emulsifiers will cause the emulsifier to form its bundle and increase the viscosity of the emulsion, thus affecting the emulsion property. In this study, the effect of emulsifier dosage on the properties of emulsion was investigated.

From Table 2, it can be seen that when the emulsifier dosage was less than 20 wt%, the stability of the produced microcrystalline wax emulsion was poor. When the emulsifier dosage exceeded 20 wt%, the stability of the emulsion was good, indicating that the emulsifier completely covered the microcrystalline wax particles and formed a complete protective film and a stable double electric layer on the surface of the microcrystalline wax particles, making the emulsion stable. However, when the emulsifier dosage continued to increase, the excess emulsifier formed micelles in the aqueous phase, which accelerated Ostwald ripening and thus affected the performance of the emulsion. As the emulsifier dosage increased, the emulsion particle size and emulsion viscosity showed a trend of first decreasing and then increasing. This was due to the microcrystalline wax particles not covered by the emulsifier colliding with each other and agglomerating at a low emulsifier dosage, thus increasing the particle size of the emulsion; and the agglomerated microcrystalline wax dispersed in the aqueous phase increased the viscosity of the emulsion. When the emulsifier was excessive, the excess emulsifier increased (*p* < 0.05) the viscosity of the emulsion, and slightly increased the particle size of the emulsion.

**Figure 2:**
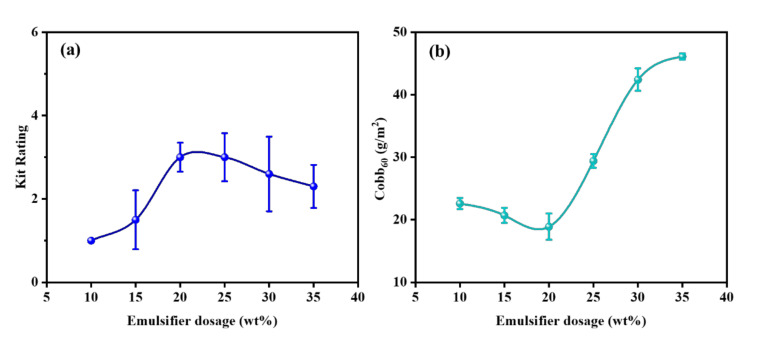
Effect of emulsifier dosage on the oil and water resistance of paper: (**a**) the kit rating value and (**b**) the Cobb_60_ value of paper.

In order to discuss the effect of emulsifier dosage on the application property of the emulsion, microcrystalline wax emulsions with different emulsifier dosages were applied to the paper surface, where the coating load was 4 g/m^2^. The results are shown in Figure 2. With the increased emulsifier dosage, the kit rating value of paper showed a trend of first increasing and then decreasing, and the Cobb_60_ value of paper showed a trend of first decreasing and then increasing. The non-stable state of the emulsion and the dosage of emulsifier affected the oil and water resistance of the paper. When the emulsifier dosage reached 20 wt%, the paper showed the best performance of oil and water resistance. As the emulsifier dosage continued to increase, the kit rating value of the paper slightly reduced, while the Cobb_60_ value of the paper increased significantly. This was due to the excessive addition of the oleophilic and hydrophilic emulsifiers, weakening the oil and water-resistant performance of the paper.

To achieve the best performance of emulsion on the and oil and water resistance of paper, the emulsifier dosage of 20 wt% was the most appropriate.

### 3.2. Microcrystalline Wax Coating

**Figure 3:**
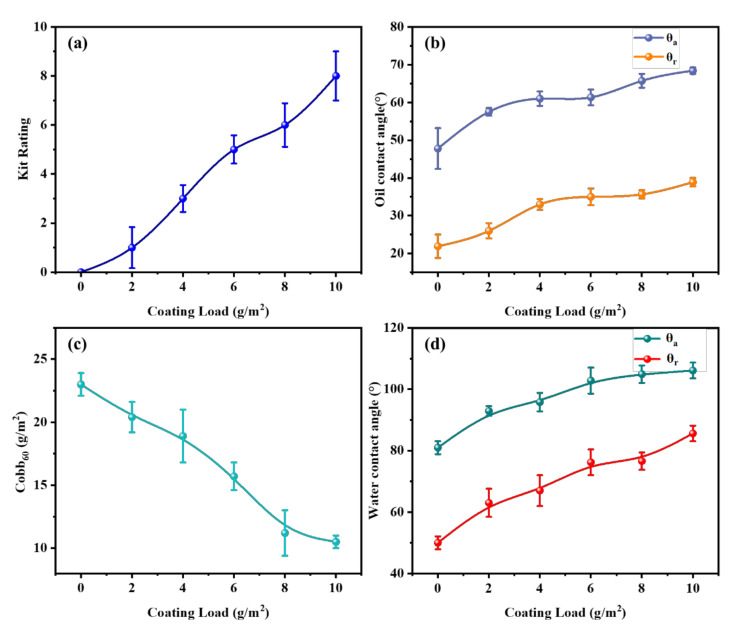
Effect of microcrystalline wax on the oil and water resistance of the original paper: (**a**) the kit rating value of paper; (**b**) the oil contact angle of paper; (**c**) the Cobb_60_ value of paper; (**d**) the water contact angle of paper. *θ_a_* is the advancing contact angle, and *θ_r_* is the receding contact angle.

The relationship between the microcrystalline wax coating load, and the oil and water resistance of paper, was investigated. In Figure 3a,b, it can be seen that the original paper showed poor oil resistance with its kit rating value of 0/12, and the advancing and receding oil contact angles were 47.8° and 21.9°, respectively. This was due to the porous structure of the paper, which caused the original paper to be easily penetrated by the grease [33,34]. The pore is one of the factors affecting the barrier properties of paper [35,36], and the microcrystalline wax effectively filled the pores on the paper surface and weakened the capillary action of the paper, thus increasing the resistance of the paper to liquids. With the increased microcrystalline wax coating load, the oil resistance of the paper was gradually enhanced, as shown by the increase in kit rating value and oil contact angle. When the microcrystalline wax coating load was increased to 10 g/m^2^, the kit rating value of paper increased to 8/12 and the advancing and receding oil contact angles were increased to 68.4° and 38.9°, respectively. Since the microcrystalline wax emulsion contained lipophilic emulsifiers, the surface treated with microcrystalline wax emulsion was easily wetted by oil, shown by the advancing and receding oil contact angles not exceeding 90°. The above data indicated that the microcrystalline wax provided a certain oil barrier for the paper; the greater the coating load, the easier it was to form an oil-resistant layer on the surface of the paper, and the better the paper oil-resistance capability.

The paper was mainly composed of fibers with hydrophilic properties, so the original paper exhibited poor water resistance. Microcrystalline wax is composed of branched saturated hydrocarbons of C31-70, which are strongly hydrophobic. By applying microcrystalline wax emulsion to the paper surface, a water-resistance layer was formed on the paper surface, which, in turn, prevented the penetration of water. As shown in Figure 3c, the Cobb_60_ value of the original paper was 23 g/m^2^, while the Cobb_60_ value of the paper with a coating load of 2 g/m^2^ was 20.4 g/m^2^, a reduction of 11.3%. With the gradually increased coating load, the Cobb_60_ value of the paper gradually decreased. When the coating load was increased to 10 g/m^2^, the Cobb_60_ value of the paper was 10.5 g/m^2^, a reduction of 54.3%. As can be seen from Figure 3d, the advancing and receding water contact angles of the original paper were 80.9° and 50.0°, respectively, indicating that the original paper could easily be wetted by water. With the gradually increased coating load, the water contact angle of the paper gradually increased. While the coating load was increased to 10 g/m^2^, the advancing and receding water contact angles of the paper rose to 106.1° and 85.6°, respectively. The addition of microcrystalline wax caused the paper to obtain a certain waterproof performance, and the larger the coating load, the better the water resistance capability of the paper.

### 3.3. Paper Pretreatment

Molecules of microcrystalline wax become disordered when melted by heat, and their mobility increases, making it easy for them to penetrate the paper through the pores of the paper surface. The phase transition temperature of the microcrystalline wax emulsion in this experiment (about 62 °C) was significantly lower than the drying temperature (Figure S1). This is why even if the microcrystalline wax coating load is large enough, it still cannot make the paper obtain excellent oil and water resistance, which obviously does not meet actual production needs. Numerous studies have shown that the combination of biopolymers and waxes for surface coating can provide excellent barrier properties for paper [20,37]. Moreover, the presence of the biopolymer coating is able to reduce the penetration of wax into the paper, thereby diminishing the weight of the wax coating [38].

**Figure 4:**
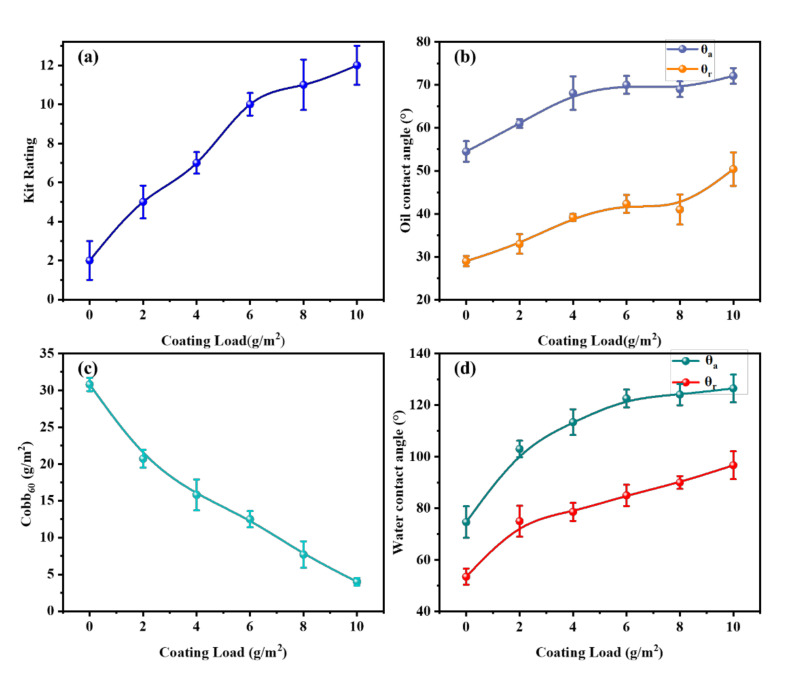
Effect of microcrystalline wax on the oil and water resistance of the pretreated paper: (**a**) the kit rating value of paper; (**b**) the oil contact angle of paper; (**c**) the Cobb_60_ value of paper; (**d**) the water contact angle of paper. *θ_a_* is the advancing contact angle, and *θ_r_* is the receding contact angle.

For further study, the paper surface was pretreated with cationic starch to compensate for the smoothness deficiency of the porous paper before microcrystalline wax coating. As shown in Figure 4, compared with the original paper (Figure 3a,c), the oil resistance of the paper coated with cationic starch only was enhanced, while its water resistance was weakened. Because of its hydrophilic nature, cationic starch negatively affected the water resistance of the paper, but it formed a barrier layer on the surface of the paper, enhancing its oil resistance. After applying microcrystalline wax to the surface of the pretreated paper, the barrier properties of the paper were mainly provided by the microcrystalline wax layer. As the coating load of microcrystalline wax increased, the oil and water resistance of the pretreated paper increased, which showed the same trend as the original paper (Figure 3). When the coating load of microcrystalline wax was 4 g/m^2^, the advancing and receding oil contact angles of the paper were 68.1° and 39.2°, respectively, and the kit rating value of paper reached 7/12, which met the requirement of oil-proof paper with a kit rating value greater than 5/12, according to the TAPPI standard. The water resistance of the paper was also greatly enhanced. The Cobb_60_ value of the paper was 15.8 g/m^2^, which was reduced by 48.7%, and the advancing and receding water contact angles of the paper rose to 113.4° and 78.6°, respectively. When the coating load of microcrystalline wax was increased to 10 g/m^2^, the paper showed excellent oil resistance with a kit rating of 12/12 and the advancing and receding oil contact angles of 72.1° and 50.4°, respectively, while the paper also showed excellent water resistance with a Cobb_60_ value of 4 g/m^2^, a reduction of 87.0%, and the advancing and receding water contact angles of 126.5° and 96.7°, respectively.

**Figure 5:**
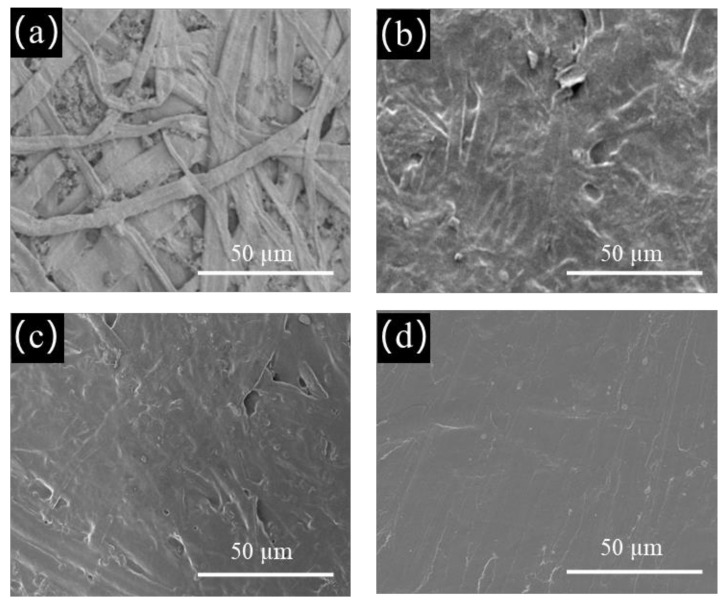
The SEM images (300×) of the: (**a**) original paper; (**b**) microcrystalline wax–coated paper with a coating load of 6 g/m^2^; (**c**) cationic starch–coated paper with a coating load of 1.5 g/m^2^; (**d**) cationic starch–microcrystalline wax–coated paper with a coating load of 6 g/m^2^.

The presence of the cationic starch coating greatly enhanced the oil and water resistance of the paper. This was because it effectively reduced the penetration of microcrystalline wax into the paper, allowing the microcrystalline wax to remain on the paper surface as much as possible, and forming a more uniform and complete barrier layer. These phenomena can be reflected in the SEM image of the paper that follows (Figure 5).

### 3.4. Surface Morphology of the Paper

This study is based on the fact that cationic starch filled some of the pores of the paper and reduced the penetration of microcrystalline wax into the paper, while microcrystalline wax enhanced the oil and water resistance of the pretreated paper. Therefore, SEM characterization was employed to observe the surface features of the original paper, microcrystalline wax–coated paper, cationic starch–coated paper, and cationic starch–microcrystalline wax–coated paper as shown in Figure 5. The fibers and pores were clearly visible on the surface of the original paper (Figure 5a). In Figure 5b, it can be seen that the surface of the microcrystalline wax–coated paper was covered by a layer of microcrystalline wax, but the pores on the paper surface still exist. After the cationic starch coating, the fibers became smoother and covered by a layer of cationic starch, and most of the pores on the paper surface were filled (Figure 5c). In Figure 5d, it can be observed that the surface of the cationic starch–microcrystalline wax–coated paper was even smoother and more uniform, and no pores can be seen. Thus, the SEM analysis well proved the vision of this study. It is clear that the pretreatment (cationic starch coating) allowed more of the microcrystalline wax to remain on the paper surface, thus enhancing the oil and water resistance of the paper.

### 3.5. Water Vapor Permeability (WVP) of the Paper

**Figure 6:**
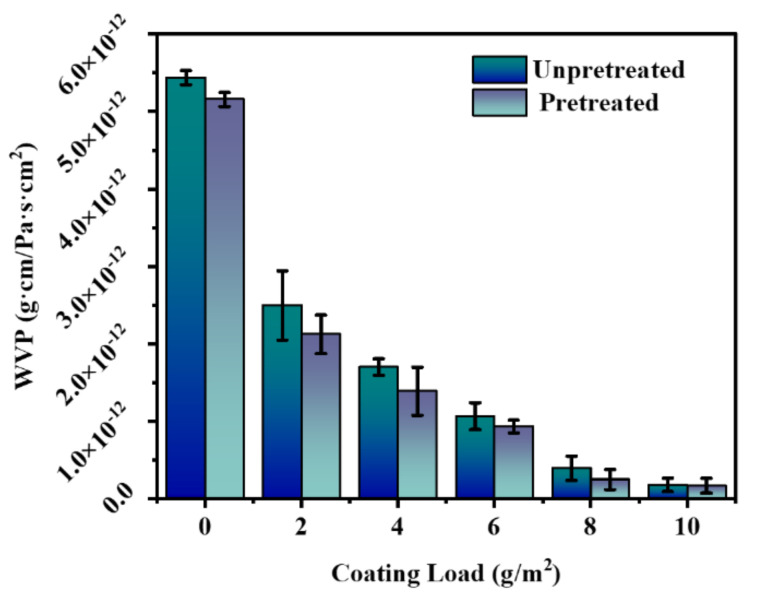
The water vapor permeability of the paper.

The WVP of the paper is shown in Figure 6. As expected, due to its porous and hydrophilic nature, the original paper exhibited poor water barrier properties with a WVP value as high as 5.436 × 10^−12^ g·cm/Pa·s·cm^2^. In contrast, the water barrier properties of the paper were significantly improved as the microcrystalline wax filled its pores and covered its surface. The WVP of the paper was 2.497 × 10^−12^ g·cm/Pa·s·cm^2^ when the microcrystalline wax coating load was 2 g/m^2^, which was reduced by 54.1%. When the microcrystalline wax coating load increased, the barrier property of paper was significantly enhanced. When the microcrystalline wax coating load was increased to 10 g/m^2^, the WVP of the coated paper was 1.816 × 10^−13^ g·cm/Pa·s·cm^2^, which was reduced by 96.7%. It was interesting to note that the WVP of the original paper was slightly reduced to 5.156 × 10^−12^ g·cm/Pa·s·cm^2^ after pretreatment. Despite its hydrophilic properties, cationic starch formed a film on the surface of the paper and filled the pores of the paper, increasing the resistance of water vapor through the paper, so the water vapor barrier performance of the pretreated paper was superior to the unpretreated paper.

Whether the paper was pretreated or not, the WVP of the paper decreased with the increased microcrystalline wax coating load. The addition of microcrystalline wax significantly enabled the paper to obtain excellent water vapor barrier properties, the higher the coating load, the better the water vapor barrier properties.

### 3.6. The Overall Migration of Paper

As a food packaging material, it is important to minimize the migration of paper components into the food. To ensure food safety, many countries and regions have issued relevant regulations, including China and the EU, which stipulate that the overall migration of packaging materials should not exceed 10 mg/dm^2^. As shown in Table 3, pretreatment does not have a large effect on the overall migration of the paper. When the microcrystalline wax coating load was 6 g/m^2^, the overall migration of the paper in the food simulant was close to 10 mg/dm^2^, and when the microcrystalline wax coating load was greater than 6 g/m^2^, the overall migration of the paper in the food simulant exceeded the limit value (*p* < 0.05). Since there are about 5% (*w*/*v*) emulsifiers in the microcrystalline wax emulsion, these emulsifiers are easily dispersed in water and ethanol, which may be the reason for the excessive migration of the paper components. Therefore, when the microcrystalline wax emulsion is applied to food packaging paper, it is recommended that the coating load of microcrystalline wax should not exceed 6 g/m^2^ for food safety.

## 4. Conclusions

The stable microcrystalline wax emulsion was successfully obtained by adjusting the emulsifier ratio and dosage by using a mechanical stirring–homogenization combination method. The application of microcrystalline wax emulsion to the paper surface provided the paper with good oil and water resistance and water vapor barrier properties. Paper pretreatment reduced the penetration of microcrystalline wax into the paper, which, in turn, diminished the weight of the wax coating. Due to the possibility of migration of paper components in food simulants, the use of microcrystalline wax emulsions should be justified for food safety, under the premise of meeting the oil and water resistance performance of paper. This study confirms that replacing fluorinated oil repellents with microcrystalline wax emulsion is a feasible solution that could bring some economic and environmental benefits to the food packaging sector.

## Figures and Tables

**Table 1:** Effect of emulsifier ratio on the performance of microcrystalline wax emulsions.

w(Span-80):w(Tween-80)	Average Particle Size/nm	Stability	HLB Value
3:7	401.9 ± 8.7 ^a^	Stratified	11.79
4:6	321.0 ± 6.5 ^b^	Unstratified	10.72
5:5	304.8 ± 5.4 ^bc^	Unstratified	9.65
6:4	316.4 ± 3.9 ^bcd^	Unstratified	8.58
7:3	369.6 ± 2.5 ^ae^	Stratified	7.51

Mean values with standard deviations. Different superscripts characters (a–e) within the same column indicate significant differences between the samples (*p* < 0.05).

**Table 2:** Effect of emulsifier dosage on the performance of microcrystalline wax emulsions.

Emulsifier Dosage (Compared to Microcrystalline Wax)/wt%	Average Particle Size/nm	Stability	Viscosity /mPa·s
10	402.9 ± 7.9 ^a^	Stratified	35.1 ± 0.7 ^a^
15	316.3 ± 2.1 ^b^	Stratified	29.7 ± 0.7 ^b^
20	304.8 ± 4.5 ^bc^	Unstratified	30.2 ± 0.4 ^bc^
25	297.2 ± 9.3 ^bcd^	Unstratified	33.5 ± 0.8 ^ad^
30	320.3 ± 1.6 ^bce^	Unstratified	48.0 ± 0.3 ^e^
35	336.1 ± 6.9 ^df^	Unstratified	57.5 ± 1.9 ^f^

Different superscript characters (a–f) within the same column indicate significant differences between the samples (*p* < 0.05).

**Table 3:** The overall migration of paper in food simulants.

Microcrystalline Wax Coating Load (g/m^2^)	The Overall Migration of Unpretreated Paper (mg/dm^2^)	The Overall Migration of Pretreated Paper (mg/dm^2^)
2	5 ± 0.8 ^a^	5.2 ± 0.4 ^a^
4	7.1 ± 1.1 ^ab^	7.5 ± 0.8 ^ab^
6	9.8 ± 0.5 ^bc^	9.4 ± 1.2 ^abc^
8	15.8 ± 2.7 ^d^	16.7 ± 1.8 ^d^
10	35.1 ± 5.4 ^e^	33.9 ± 4.7 ^e^

Different superscript characters (a–e) within the same column indicate significant differences between the samples (*p* < 0.05).

## Data Availability

The data presented in this study are available on request from the corresponding author.

## Supplementary Materials

The following supporting information can be downloaded at: https://www.mdpi.com/article/10.3390/polym14091786/s1, Figure S1: Thermal analyses of microcrystalline wax and dried microcrystalline wax emulsion; (a) thermalgravimetric analysis (TGA), (b) derivative thermogravimetry (DTG), (c) differential scanning calorimeter (DSC).
