# Supplementary material for: Improvement of Oil and Water Barrier Properties of Food Packaging Paper by Coating with Microcrystalline Wax Emulsion
Source: Polymers (Basel). 2022 Apr 27;14(9):1786. doi: 10.3390/polym14091786 (PMC9099909; doi:10.3390/polym14091786)
Supplement: Supplementary file 1 [file polymers-14-01786-s001.zip › polymers-1689695-supplementary.pdf]

polymers

MDPI

Supplementary materials: Improvement of Oil and Water Barrier Properties of Food Packaging Paper by Coating with Microcrystalline Wax Emulsion

Dongyang Liu, Yuqing Duan, Shumei Wang, Murong Gong and Hongqi Dai*

Jiangsu Co-Innovation Center of Efficient Processing and Utilization of Forest Resources, Nanjing Forestry University, Nanjing, 210037, China; dyliu@njfu.edu.cn (D.L.)

* Correspondence: hgdhq@njfu.edu.cn; Tel: 13605174149 (H.D.)

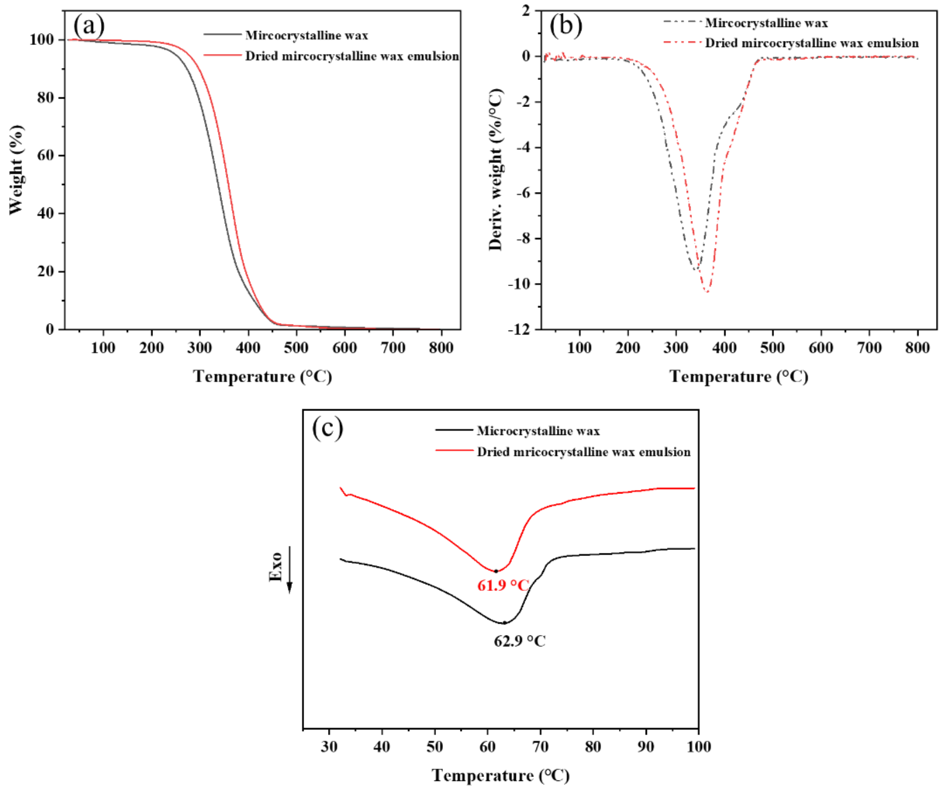

Figure S1. Thermal analyses of microcrystalline wax and dried microcrystalline wax emulsion; (**a**) thermalgravimetric analysis (TGA), (**b**) derivative thermogravimetry (DTG), (**c**) differential scanning calorimeter (DSC).
